# Regulatory T cells differentiation in visceral adipose tissues contributes to insulin resistance by regulating JAZF‐1/PPAR‐γ pathway

**DOI:** 10.1111/jcmm.17680

**Published:** 2023-02-03

**Authors:** Fanping Meng, Po Hao, Hongxin Du

**Affiliations:** ^1^ Department of Medical Laboratory Chongqing University Three Gorges Hospital Chongqing China; ^2^ Department of Medical Technology Chongqing Three Gorges Medical College Chongqing China

## Abstract

Regulatory T cell (Treg) activity and differentiation in visceral adipose tissue (VAT) play an important role in inhibiting chronic inflammation and insulin resistance. Whether JAZF‐1 and PPAR‐γ mediate VAT Treg differentiation to promote the inhibition of chronic inflammation and insulin resistance remains unclear. Here, we investigated the roles of JAZF‐1 and PPAR‐γ in VAT Treg differentiation, inflammation and insulin resistance using a transgenic mouse model. First, we determined that the levels of glucose and insulin biochemical markers in the JAZF‐1 transgenic general feeding or high‐fat groups were lower than those in the wild‐type general feeding or high‐fat groups. Second, the levels of CD4^+^, CD25^+^, and FOXP3^+^ differentiation markers in the JAZF‐1 transgenic general feeding or high‐fat groups were significantly higher than those in the wild‐type groups. PPAR‐γ inhibition was associated with low levels of CD4^+^, CD25^+^ and FOXP3^+^ differentiation markers. Third, the levels of TNF‐α, IL‐1β and IL‐6 in the JAZF‐1 transgenic groups were lower than those in the wild‐type groups, whereas IL‐10 and TGF‐β levels were higher in the JAZF‐1 transgenic groups than in the wild‐type groups. After using the PPAR‐γ inhibitor, we observed that TNF‐α, IL‐1β and IL‐6 increased, while IL‐10 and TGF‐β decreased. We found that JAZF‐1 and PPAR‐γ could promote Tregs differentiation and regulate insulin resistance by synergistically decreasing the expression levels of TNF‐α, IL‐1β and IL‐6 and increasing those of IL‐10 and TGF‐β.

## 1 INTRODUCTION

Insulin resistance is characterized by normal circulating insulin with decreased responsiveness to target tissues, which can cause persistent production of insulin and subsequent secondary hyperinsulinemia.^1^, ^2^ Studies have demonstrated that insulin resistance can be induced by abnormal body fat distribution or obesity.^3^, ^4^ Furthermore, insulin resistance is significantly associated with the risk of type 2 diabetes.^5^ Adipocytes are one of the most sensitive cells to insulin, but free fatty acids can weaken the body's sensitivity to insulin, leading to insulin resistance.^6^ Moreover, the inflammatory response induced in mature adipocytes is regulated by their protein secretion that can be controlled by the expression of enzymes.^7^ Small vesicles secreted by adipose tissue wrap adipokines, mediate macrophage activation, promote inflammation and lead to insulin resistance. Understanding the mechanisms underlying the progression of insulin resistance can lead to the development of new strategies that relieve insulin resistance and subsequent metabolic diseases.

Local inflammation can occur in visceral adipose tissue (VAT) with the secretion of various fat‐specific inflammatory factors and is involved in glucose and lipid metabolism.^8^ Regulatory T cells (Tregs) express CD4, CD25 and forkhead box P3 (FOXP3), inhibiting immune and inflammatory responses.^9^ Numerous Tregs in VAT can suppress the inflammatory response by secreting cytokines.^10^ Moreover, a reduction in the number of Tregs can give rise to metabolic disorders.^11^, ^12^ Therefore, regulating the differentiation and activity of VAT Tregs plays an essential role in inhibiting chronic inflammation and insulin resistance.

Peroxisome proliferator‐activated receptor gamma (PPAR‐γ) is a proliferator‐activated receptor in adipose tissues with anti‐inflammatory properties and is an important transcription factor that regulates fat remodelling and macrophage polarization.^13^ PPAR‐γ activates the transcription of multiple genes and regulates adipocyte, T cell, and macrophage functions. A previous study demonstrated that PPAR‐γ selective knockout in macrophages is significantly related to insulin resistance progression.^14^ PPAR‐γ is considered a key factor for regulating Tregs in adipose tissue, promoting the differentiation and activity of VAT Tregs.^15^, ^16^ PPAR‐γ can affect the inflammatory response by regulating T cells from the early stages of inflammation into the antioxidant stage.^17^

In addition, juxtaposed with another zinc finger gene 1 (JAZF‐1) is highly expressed in adipose tissues and is involved in regulating gluconeogenesis, insulin sensitivity, lipid metabolism and inflammatory responses.^18^ Studies have demonstrated that JAZF‐1 polymorphism is significantly associated with the progression of type 2 diabetes,^19^ and JAZF‐1 overexpression enhances glucose tolerance and insulin sensitivity.^20^ However, the role of JAZF‐1 and its downstream pathway molecules in Treg differentiation remains unclear. Therefore, this study aimed to assess the mechanism of action of JAZF‐1 and PPAR‐γ on the progression of insulin resistance in VAT Tregs. We found that JAZF‐1 and PPAR‐γ could activate Treg differentiation in VAT. These proteins also may work together to decrease the expression levels of TNF‐α, IL‐1β and IL‐6, and increase those of IL‐10 and TGF‐β. Altogether, we believe that JAZF‐1 and PPAR‐γ function synergistically to reduce insulin resistance in VATs.

## 2 MATERIALS AND METHODS

### 2.1 Chemicals, reagents and antibodies

The adeno‐associated virus (AAV) helper‐free system was purchased from Agilent Technologies (catalog # 240071), and the pAAV‐JAZF‐1 plasmid construction followed the AAV helper‐free system instructions. After amplifying and extracting the endotoxin‐free plasmid, pAAV‐JAZF‐1 was transfected into AAV‐293 cells. After the cells became large, round, and floated like grape clusters, the virus was collected and purified by caesium chloride (CsCl) density gradient centrifugation. The viral concentration was determined by quantitative PCR (qPCR), and the number of copies was calculated. After packaging, the virus was suspended in 4% sucrose buffer and stored in a refrigerator at −80°C.

### 2.2 Animal experiments

Male C57BL/6 wild‐type mice (4 weeks old, weighing 15 ± 3 g, SPF grade) were purchased from Chongqing Enswell Biotechnology Co., LTD (Chongqing, China). All protocols in this study were approved by the Laboratory Animal Care Committee of Three Gorges Hospital, affiliated with Chongqing University. The mice were fed adaptively for 2–12 h, alternating day and night, temperature of 20–22°C, humidity of 40%–60%, drinking water freely and eating ordinary feed. After 1 week, 32 mice were randomly divided into wild‐type general feeding, wild‐type high‐fat diet, JAZF‐1 transgenic general feeding and JAZF‐1 transgenic high‐fat diet groups (*n* = 8). The weight and energy compositions between the general diet and high‐fat diet are shown in Table 1. Transgenic mice overexpressing JAZF‐1 were obtained by continuously injecting AAV‐JAZF‐1 in their tail vein for 3 days. Another 32 mice were injected with AAV‐JAZF‐1 and randomly assigned to general feeding plus normal saline, high‐fat diet plus normal saline, general feeding plus PPAR‐γ inhibitor (GW9662) and high‐fat diet plus PPAR‐γ inhibitor (*n* = 8). The insulin resistance model was constructed using a high‐fat diet, while an ordinary diet was given to the control group, and the body weight of mice was recorded weekly.

**TABLE 1:** Comparison of the weight and energy compositions between the general diet and high‐fat diet.

Nutritional ingredient	General diet (%)		High‐fat diet (%)	
	Weight (wt/wt)	Energy (kJ/kJ)	Weight (wt/wt)	Energy (kJ/kJ)
Protein	18.81	21	13.14	13
Fat	7.39	19	28.49	54
Carbohydrate	51.96	60	40.87	33
Other	21.94	—	17.5	—
Total	100	100	100	100

### 2.3 Biochemical markers

After 12 weeks, the mice from all eight groups fasted for 12 h and intraperitoneally injected with 2 g/kg glucose. Peripheral blood was collected from the tail vein at 0, 15, 30, 60 and 120 min. Insulin and glucose tolerance were measured using an insulin tolerance test (ITT) and glucose tolerance test (GTT) respectively. Moreover, total cholesterol (TC), triglyceride (TG) and glucose (GLU) levels in the blood were measured using an E600 automatic biochemical analyzer.

### 2.4 CD4^+^ T cells in peripheral blood and Tregs differentiation

For the anticoagulation test, blood from the tail vein of mice from each of the eight study groups was taken and diluted with an equal volume of tissue diluent before centrifugation. The white membrane layer (CD4^+^ T cells) was centrifuged and washed, and the cells were suspended in Iscove's modified Dulbecco's medium (IMDM) containing 10% foetal bovine serum (FBS), 2 mM glutamine, 100 U/mL penicillin, 100 μg/mL streptomycin and 50 μM β‐mercaptoethanol. Live cells were counted, and CD4^+^ T cells were sorted by flow cytometry. The flow rate and sample concentration were adjusted according to the content of CD4^+^ T cells such that the concentration of CD4^+^ T cells through the sample chamber was 200 s^−1^. The cells obtained were centrifuged and concentrated at 300 × *g* for 5 min. The IMDM was used to re‐suspend and transfer the cells to an incubator at 37°C with 5% CO_2_. One day before induction, a phosphate buffered saline (PBS) solution containing 5 μg/mL anti‐mouse CD28 and anti‐mouse CD3 antibodies was added to the Petri dish and coated overnight at 4°C. The next day, CD4^+^ T cells were added to the pre‐coated Petri dishes. Meanwhile, 100 nM recombinant IL‐2 was added to induce differentiation. Three days later, differentiation rate peak was reached, and the following experiments were performed. Mice cells from each one of the eight study groups were evenly divided into three tubes for immunofluorescence staining. Anti‐mouse CD4‐FITC, anti‐mouse CD25‐APC and anti‐mouse FOXP3‐PE were added to all the eight group tubes, and the Treg cells differentiation status was detected by flow cytometry.

### 2.5 Inflammatory markers

For all eight study groups, we collected the serum of the mice's tail vein or the homogenate supernatant of peritoneal omental adipocytes. PBS was added to process the tissue samples into a single‐cell suspension. The cytokines in the serum or tissue homogenate supernatant were detected, including TNF‐α, IL‐1β, IL‐6, IL‐10 and TGF‐β. The flow cytometry was performed according to the operating procedures of the cytometric bead array (CBA) kit.

### 2.6 JAZF‐1 and PPAR‐γ expression and their interaction

After 12 weeks of differential feeding, mice in each group were killed by cervical dislocation, and 70% chloral hydrate was injected intraperitoneally to open the abdominal cavity and separate the adipose tissue of the mice. The mRNA and protein levels of JAZF‐1 and PPAR‐γ were detected by RT‐qPCR and western blotting respectively. PCR primer sequences are listed in Table 2. The cell samples were washed 1–3 times with PBS, and 1 mL RNAiso Plus lysate was added, while the RIPA lysate was added for total protein extraction. The concentration of RNA were calculated by the absorption peaks and ratios of 230, 260 and 280 nm in trace spectrophotometer (Thermo, USA; model number: NanoDrop One/One C). The interaction between JAZF‐1 and PPAR‐γ was detected using an immunoprecipitation (Co‐IP) assay in a Co‐IP kit following the manufacturer's instructions (Basin Biotechnology, Inc. catalog # Bes 3011). CD4^+^ T cells were isolated from peripheral blood lymphocytes of wild‐type mice and JAZF1 transgenic mice by flow cytometry. Cells were cultured in IMDM (Invitrogen) medium containing 2 mM L‐glutamine, 100 U/mL penicillin, 100 μg/mL streptomycin, 50 μM β‐mercaptoethanol and 10% FBS. Wild‐type mice was regarded as control group, while JAZF1 transgenic mice was defined in intervention. PPAR‐γ agonist was rosiglitazone, with EC_50_ of 60 nM, while PPAR‐γ inhibitor was GW9662, with IC_50_ of 3.3 nM. When the cells were lysed under non‐denatured conditions, the PPAR‐γ‐JAZF1 interaction in intact cells was preserved, and the PPAR‐γ‐JAZF1 protein complex was detected by Western blot.

**TABLE 2:** Primer sequences for RT‐qPCR (β‐actin).

Gene	Sequence (5′ to 3′)
JAZF‐1	
Forward	TTCTTCTCCAACACCTGCCGC
Reverse	CGATGTGGTTGTCCTCGATGTG
PPAR‐γ	
Forward	CCTGCAGGAGCAGAGCAAAGA
Reverse	ACTTGAGCAGAGTCACTTGGTCATT
GAPDH	
Forward	AGGTCGGTGTGAACGGATTTG
Reverse	TGTAGACCATGTAGTTGAGGTCA

### 2.7 Statistical analyses

SPSS software (version 22.0; SPSS Inc., Chicago, IL, USA) was used to conduct the statistical analyses. All data are shown as mean ± standard deviation (SD), and the differences among groups were assessed using one‐way analysis of variance (anova), and Tukey's post hoc test was applied to perform pair comparisons. The inspection level was two sided, and statistical significance was set at *p* < 0.05.

## 3 RESULTS

### 3.1 JAZF‐1 promotes insulin sensitivity

**FIGURE 1:**
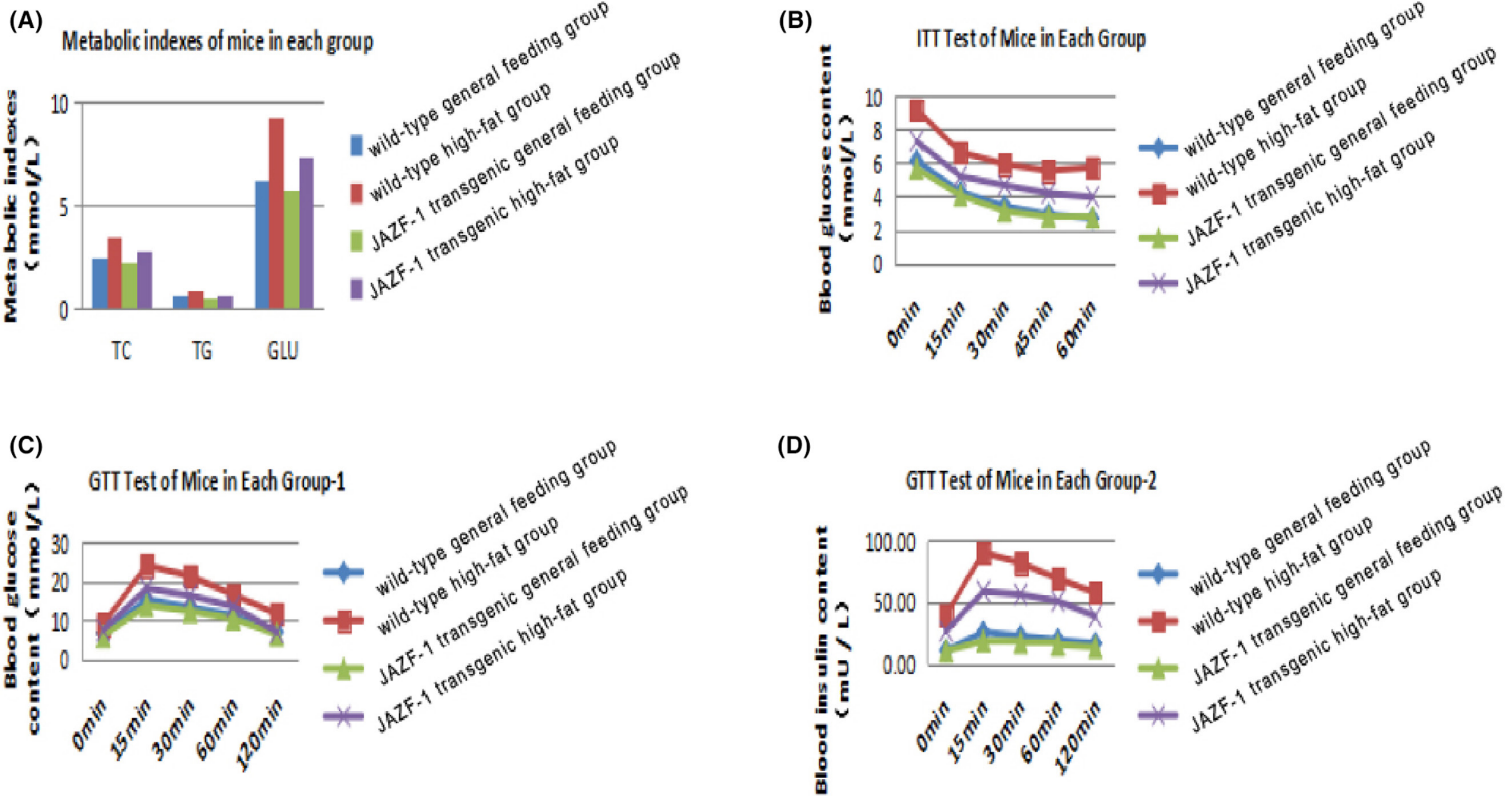
The biochemical markers in the wild‐type general feeding, wild‐type high‐fat, JAZF‐1 transgenic general feeding and JAZF‐1 transgenic high‐fat groups. (A) TC, TG and GLU levels in the wild‐type general feeding, wild‐type high‐fat, JAZF‐1 transgenic general feeding and JAZF‐1 transgenic high‐fat groups; (B) Blood glucose content assessed by ITT test in the wild‐type general feeding, wild‐type high‐fat, JAZF‐1 transgenic general feeding and JAZF‐1 transgenic high‐fat groups; (C) Blood glucose content assessed by GTT test in the wild‐type general feeding, wild‐type high‐fat, JAZF‐1 transgenic general feeding, and JAZF‐1 transgenic high‐fat groups; (D) Blood insulin content assessed by GTT test in the wild‐type general feeding, wild‐type high‐fat, JAZF‐1 transgenic general feeding and JAZF‐1 transgenic high‐fat groups.

The biochemical markers in the wild‐type general feeding, wild‐type high‐fat, JAZF‐1 transgenic general feeding and JAZF‐1 transgenic high‐fat groups are shown in Figure 1 and Appendix S1. We noted that the weight graduaally increased from 1 to 12 week, and the JAZF‐1 transgenic group were associated with lower weight when compared with the wild‐type groups at various time points (Figure S1 in Appendix S1). The TC, TG and GLU levels in high‐fat group was significantly increased, while JAZF‐1 transgenic mice were associated with lower TC, TG and GLU levels (Figure 1A). Moreover, the ITT test indicated that the blood glucose content was reduced after insulin injection, and that it was lower in the JAZF‐1 transgenic general feeding or high‐fat groups than that in the wild‐type general feeding or high‐fat groups at various time points respectively (Figure 1B). Furthermore, the GTT test indicated that blood glucose and insulin contents were significantly increased within 15 min after glucose injection that were shown to be reduced at later time points. The blood glucose and insulin contents in the JAZF‐1 transgenic groups were lower than those in the wild‐type groups at various time points (Figure 1C,D).

### 3.2 JAZF‐1 and PPAR‐γ regulate VAT Tregs differentiation

**FIGURE 2:**
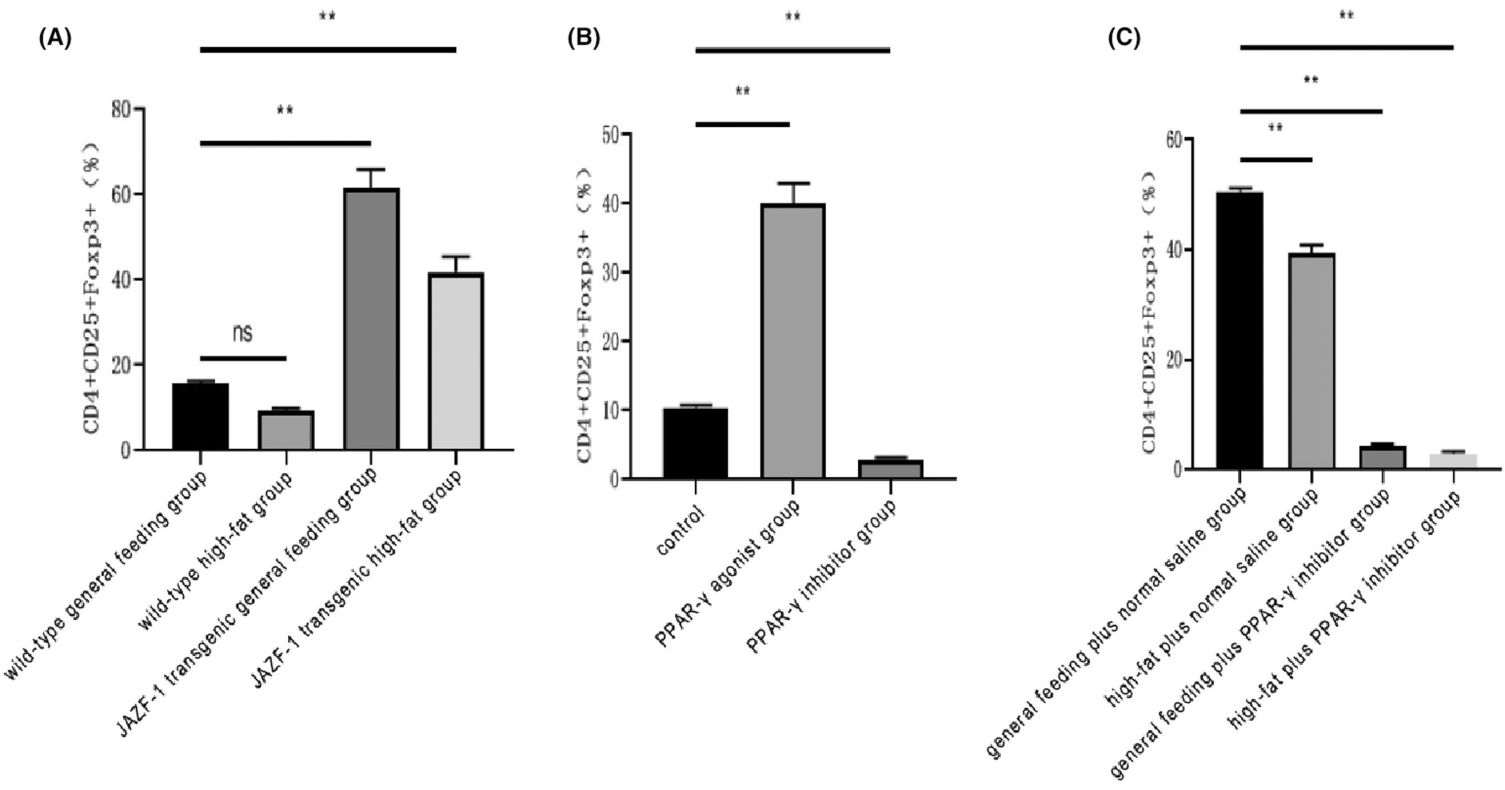
Treg differentiation in VAT was assessed using CD4^+^, CD25^+^ and FOXP3^+^ antibodies. (A) CD4^+^, CD25^+^ and FOXP3^+^ in wild‐type general feeding, wild‐type high‐fat, JAZF‐1 transgenic general feeding, and JAZF‐1 transgenic high‐fat groups; (B) CD4^+^, CD25^+^ and FOXP3^+^ in control (JAZF‐1 transgenic), PPAR‐γ agonist, and PPAR‐γ inhibitor groups for AAV‐Jazf1 mice; (C) CD4^+^, CD25^+^ and FOXP3^+^ in general feeding plus normal saline, high‐fat plus normal saline, general feeding plus PPAR‐γ inhibitor and high‐fat plus PPAR‐γ inhibitor groups for AAV‐Jazf1 mice.

Treg differentiation in VAT was assessed using CD4^+^, CD25^+^ and FOXP3^+^ antibodies, and the results are shown in Figure 2 and Appendix S2 (Figures S1–S3). By comparing the same feeding treatments, we observed a higher percentage of VAT Tregs in the JAZF‐1 transgenic groups than that in the wild‐type groups. These results suggest that JAZF‐1 promotes Treg differentiation in VAT (Figure 2A). To assess the role of PPAR‐γ on VAT Tregs, we noted the percentage of VAT Tregs in the JAZF‐1 transgenic high‐fat diet PPAR‐γ agonist group was significantly higher than that in the PPAR‐γ inhibitor and control groups, while the PPAR‐γ inhibitor group was associated with a lower percentage of VAT Tregs than the control group (Figure 2B). Finally, the percentage of VAT Tregs in PPAR‐γ inhibitor groups was significantly lower than that in each respective control group treated with normal saline (Figure 2C).

### 3.3 JAZF‐1 and PPAR‐γ affect inflammatory markers expression

**FIGURE 3:**
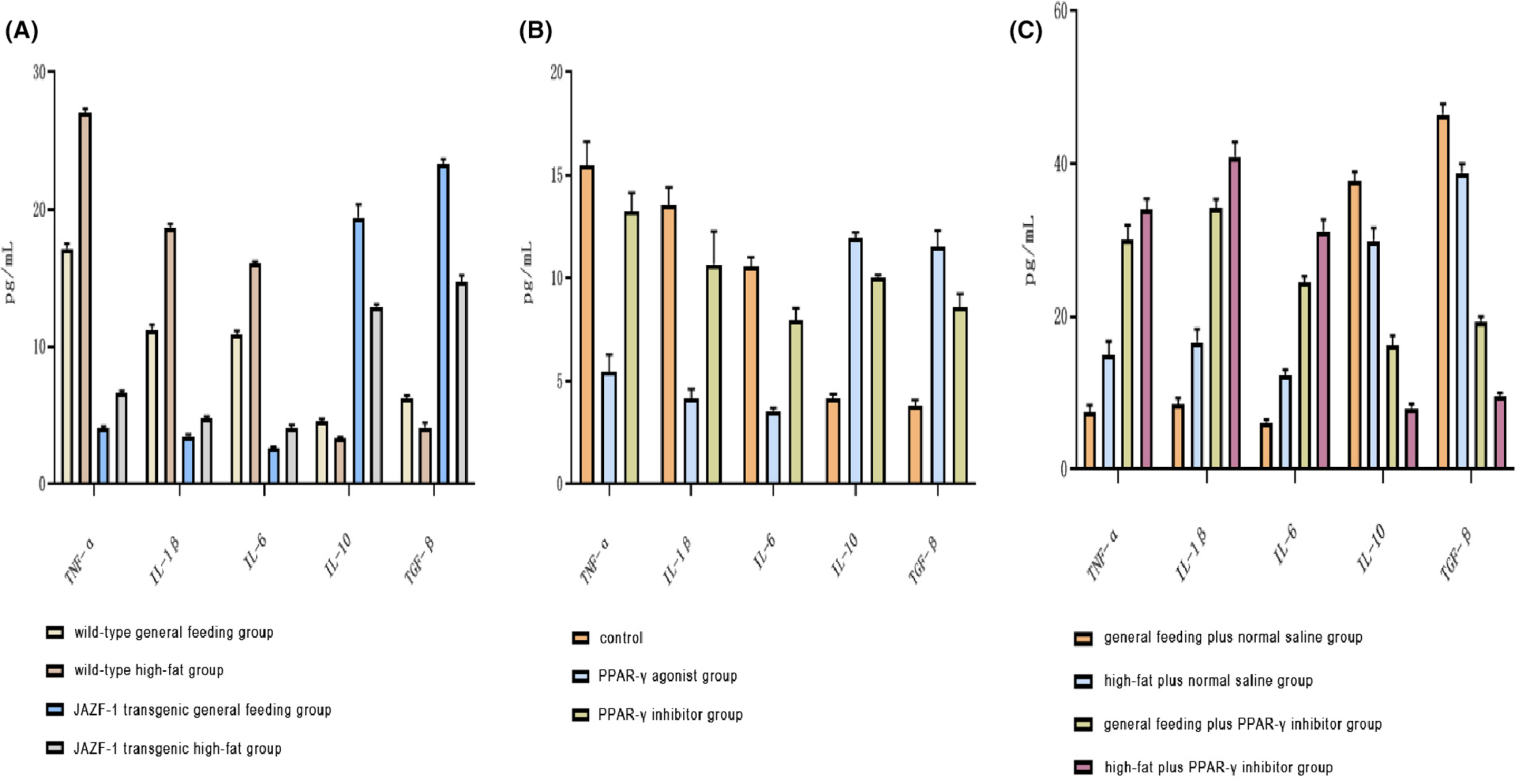
The TNF‐α, IL‐1β, IL‐6, IL‐10 and TGF‐β levels in each group. (A) TNF‐α, IL‐1β, IL‐6, IL‐10 and TGF‐β levels in wild‐type general feeding, wild‐type high‐fat, JAZF‐1 transgenic general feeding, and JAZF‐1 transgenic high‐fat groups; (B) TNF‐α, IL‐1β, IL‐6, IL‐10 and TGF‐β levels in control (JAZF‐1 transgenic), PPAR‐γ agonist, and PPAR‐γ inhibitor groups; (C) TNF‐α, IL‐1β, IL‐6, IL‐10 and TGF‐β levels in general feeding plus normal saline, high‐fat plus normal saline, general feeding plus PPAR‐γ inhibitor and high‐fat plus PPAR‐γ inhibitor groups.

The TNF‐α, IL‐1β, IL‐6, IL‐10 and TGF‐β levels in each group are shown in Figure 3 and the Appendix [Link], [Link]. We observed that TNF‐α, IL‐1β and IL‐6 levels in the JAZF‐1 transgenic general feeding group were lower than those in the corresponding wild‐type group. Similarly, the JAZF‐1 transgenic high‐fat group showed lower TNF‐α, IL‐1β and IL‐6 levels as compared to those in the wild‐type high‐fat group. Contrarily, IL‐10 and TGF‐β levels in the JAZF‐1 transgenic general feeding and high‐fat groups were significantly higher than those in the wild‐type general feeding and high‐fat groups respectively (Figure 3A). Furthermore, we noted that TNF‐α, IL‐1β and IL‐6 levels in the JAZF‐1 transgenic PPAR‐γ agonist group were significantly lower than those in the PPAR‐γ inhibitor and control groups. However, the levels of IL‐10 and TGF‐β in the PPAR‐γ agonist group were higher than those in the PPAR‐γ inhibitor and control groups (Figure 3B). Finally, TNF‐α, IL‐1β and IL‐6 levels in the JAZF‐1 transgenic general or high‐fat diets plus PPAR‐γ inhibitor groups were significantly lower than those in the general or high‐fat diet control groups respectively. However, the IL‐10 and TGF‐β levels were significantly higher in mice fed with general or high‐fat diets and PPAR‐γ inhibition than those in the respective control groups with the same feeding and without PPAR‐γ inhibition (Figure 3C).

### 3.4 Co‐expression and interaction of JAZF‐1 and PPAR‐γ

**FIGURE 4:**
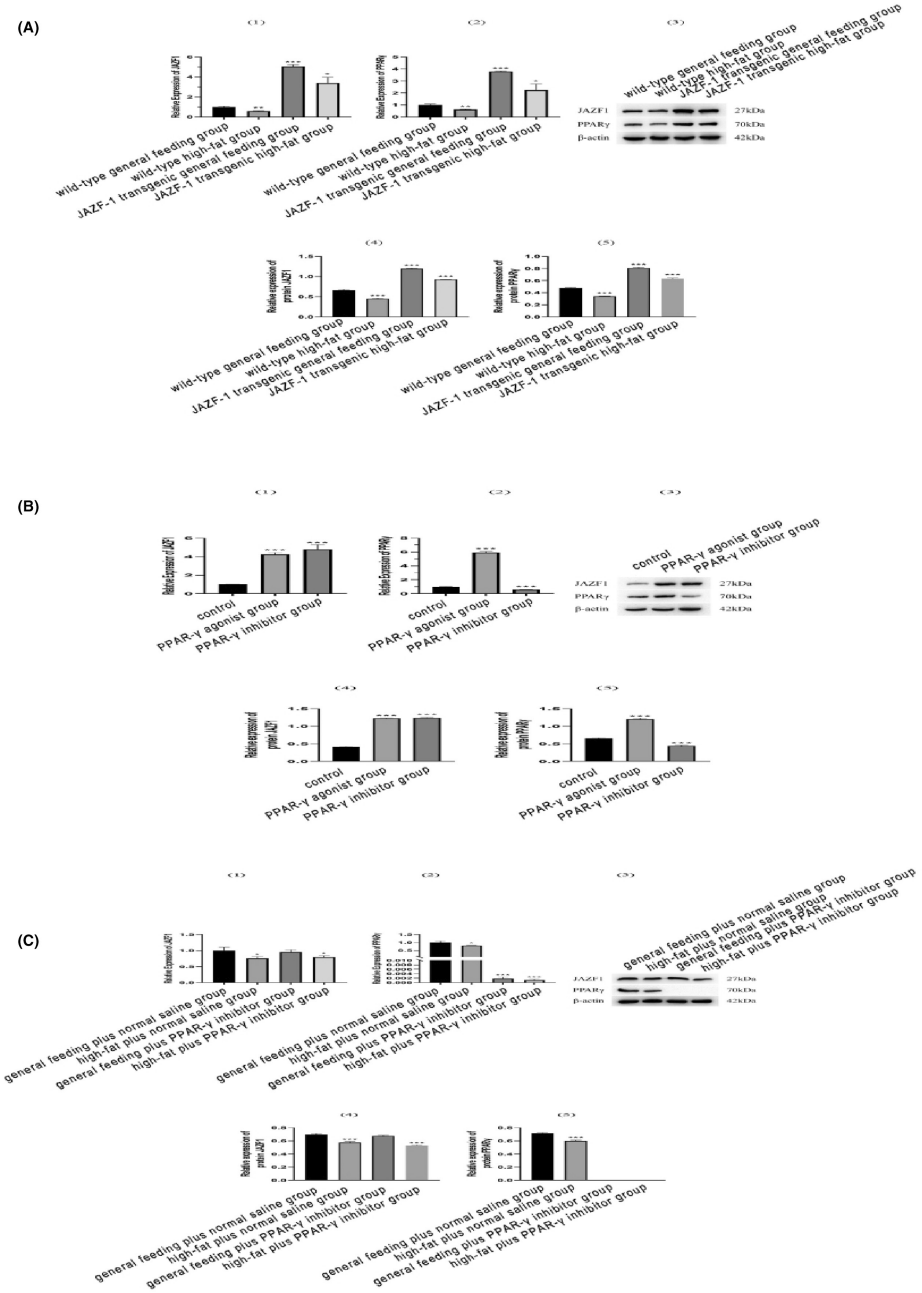
The mRNA and protein expression levels of JAZF‐1 and PPAR‐γ. (A) The mRNA and protein expression levels of JAZF‐1 and PPAR‐γ in wild‐type general feeding, wild‐type high‐fat, JAZF‐1 transgenic general feeding, and JAZF‐1 transgenic high‐fat groups; (B) The mRNA and protein expression levels of JAZF‐1 and PPAR‐γ in control (JAZF‐1 transgenic), PPAR‐γ agonist and PPAR‐γ inhibitor groups; (C) The mRNA and protein expression levels of JAZF‐1 and PPAR‐γ in general feeding plus normal saline, high‐fat plus normal saline, general feeding plus PPAR‐γ inhibitor and high‐fat plus PPAR‐γ inhibitor groups.

**FIGURE 5:**
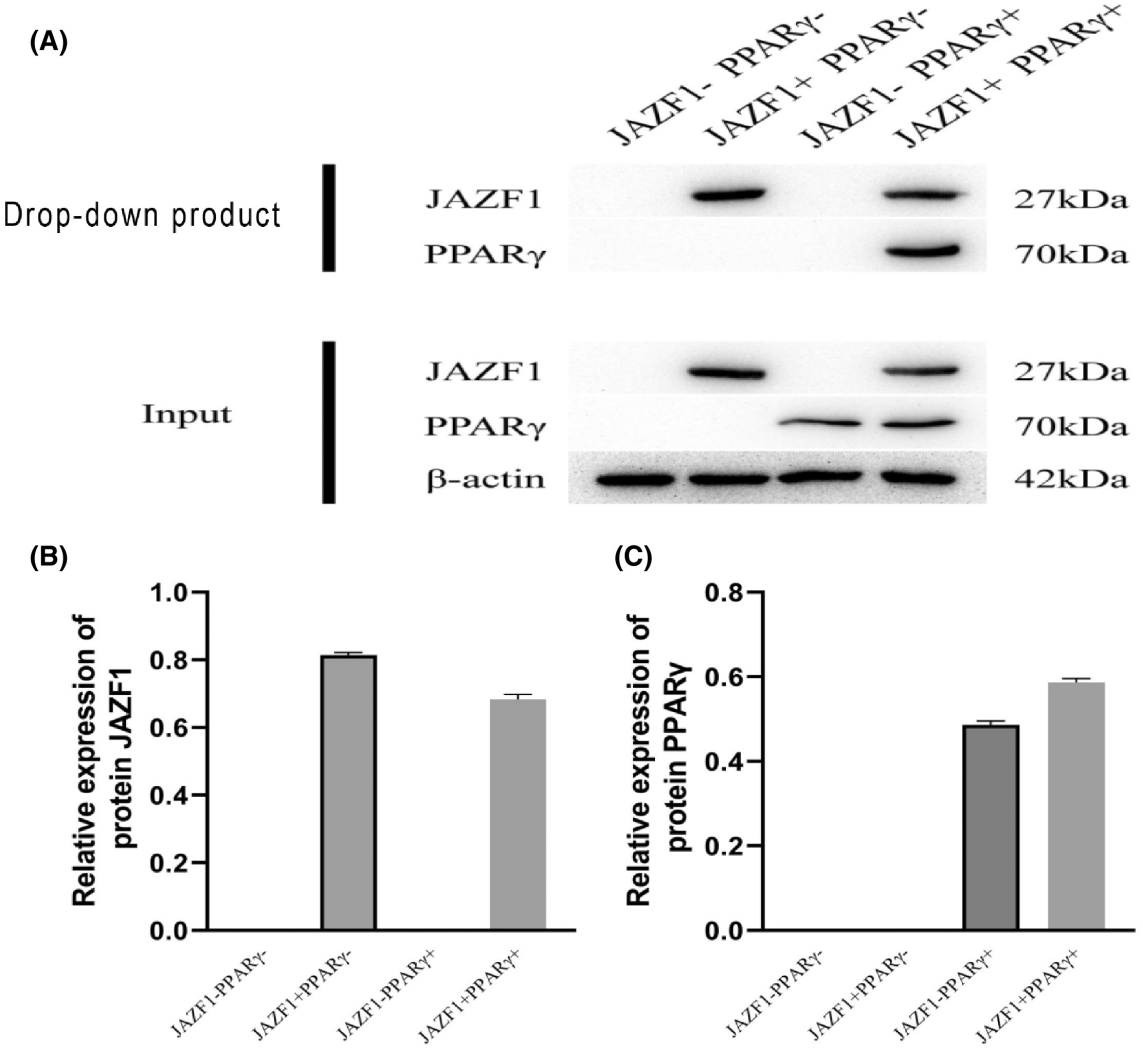
The interaction between JAZF‐1 and PPAR‐γ proteins. (A) The interaction between JAZF‐1 and PPAR‐γ proteins tested by western blotting; (B) The relative expression of JAZF‐1 protein; (C) The relative expression of PPAR‐γ protein (PPAR‐γ+ JAZF1−, and PPAR‐γ− JAZF1− groups were defined as control group, while PPAR‐γ+ JAZF1+ and PPAR‐γ− JAZF1+ were defined as intervention group; PPAR‐γ inhibitor was GW9662 and PPAR‐γ agonist was rosiglitazone).

The mRNA and protein expression levels of JAZF‐1 and PPAR‐γ are shown in Figure 4. As expected, the JAZF‐1 transgenic mice showed upregulation of JAZF‐1 mRNA and protein when compared to the wild‐type general feeding mice, independent of the feeding treatment. PPAR‐γ was also induced in JAZF‐1 transgenic mice, suggesting a potential synergistic effect between JAZF‐1 and PPAR‐γ (Figure 4A and Figures S1–S9 in Appendix S3). Moreover, the JAZF‐1 and PPAR‐γ mRNA and protein expression levels in the JAZF‐1 transgenic mice agonist to PPAR‐γ were significantly higher than those in the control group. Notably, the PPAR‐γ inhibitor was associated with low PPAR‐γ expression (Figure 4B). These results encourage the hypothesis of synergistic function between JAZF‐1 and PPAR‐γ. Furthermore, PPAR‐γ mRNA and protein expression in mice under general diet or high‐fat were significantly lower in the PPAR‐γ inhibitor group than those in the respective normal saline groups (Figure 4C). Finally, the interaction between JAZF‐1 and PPAR‐γ proteins was further investigated, and co‐IP results did not find interactions between JAZF‐1 and PPAR‐γ (Figure 5 and Tables S1 and S2 in Appendix S4).

## 4 DISCUSSION

In this study, we investigated the mechanism of action of JAZF‐1 and PPAR‐γ in VAT Tregs differentiation and inflammation during the progression of insulin resistance using a mouse model. High‐fat diet induced insulin resistance in male C57BL/6 wild‐type mice and was used to examine the effect of JAZF‐1 and PPAR‐γ on VAT Tregs. The results in the current study showed that TC, TG and GLU in the JAZF‐1 transgenic groups were lower than those in the respective wild‐type groups. These results were verified and expanded in the ITT and GTT tests. Moreover, Treg differentiation in VAT could be activated by JAZF‐1 and promoted by the PPAR‐γ. Furthermore, JAZF‐1 inhibited TNF‐α, IL‐1β or IL‐6 and promoted IL‐10 and TGF‐β expressions. Interestingly, PPAR‐γ activity was not evident in relation to cytokines regulation, as both PPAR‐γ agonism and inhibition repressed TNF‐α, IL‐1β and IL‐6 and promoted IL‐10 and TGF‐β expressions. Finally, PPAR‐γ expression was activated in JAZF‐1 overexpressing mice, as well as a functional PPAR‐γ was associated with high JAZF‐1 expression level, suggesting a potential synergistic effect between JAZF‐1 and PPAR‐γ.

Initially, PPAR‐γ assumed a transcriptional role focused on fat‐cell differentiation. A previous study has shown that PPAR‐γ expression in Tregs is significantly related to insulin sensitivity.^15^ Moreover, PPAR‐γ expression plays an important role in macrophages,^21^, ^22^ muscles,^23^ and the central nervous system.^24^, ^25^ The potential reasons for PPAR‐γ functioning across a wide range of cell types are as follows: (1) thiazolidinediones could act upstream insulin resistance, including ingestive behaviour, adiposity and inflammation; (2) insulin resistance has organ‐specific effects that abrogate PPAR‐γ expression in different cell types;^23^, ^24^, ^25^ and (3) the transgenic mouse lines expressing PPAR‐γ with cell‐type‐specific promoters may still generate unwanted transgene expression in other cell types.

Our study found that JAZF‐1 could decrease the levels of TC, TG and GLU, which could be explained by the transcriptional regulatory function of JAZF‐1, interacting with various nuclear receptors and protein kinases involved in cellular energy metabolism, including inflammatory response and glucose/lipid metabolism.^26^ Moreover, rs849334 of JAZF‐1 is significantly associated with insulin clearance, which could predict the progression of diabetes.^27^ In our results, Treg differentiation could be activated by JAZF‐1 and PPAR‐γ, and PPAR‐γ inhibitors could inhibit Treg differentiation, consistent with previous studies.^28^, ^29^ The PPAR‐γ signalling pathway plays an essential role in regulating various biological processes, especially in the liver.^30^ The proliferation and differentiation of adipose tissues can be activated by PPAR‐γ signalling.^31^ PPAR‐γ agonists can improve hepatic steatosis and liver lesions,^32^ while PPAR‐γ inhibitors significantly increase fat cell necrosis.^33^ Furthermore, JAZF‐1 can prevent lipogenesis and systematic inflammatory response, which play a role in glucose homeostasis, enhancing glucose tolerance and insulin sensitivity.^20^, ^34^

The expression of inflammatory cytokines was inhibited by JAZF‐1 and PPAR‐γ, while JAZF‐1 and PPAR‐γ activated IL‐10 and TGF‐β inhibitory cytokines. A previous study has demonstrated that Tregs regulate immune responses and have a protective role in hepatic damage.^35^ Moreover, JAZF‐1 could promote macrophage polarization from the M1 to M2 phenotype and increase the number of Tregs, which could induce the production of anti‐inflammatory cytokines.^20^ Furthermore, PPAR‐γ downregulates innate and adaptive immune cells, and PPAR‐γ expression is associated with reduced inflammation and hyperresponsiveness.^36^, ^37^, ^38^

Our study found a potential synergistic effect between JAZF‐1 and PPAR‐γ. The possible mechanism may involve PPARs binding to PPAR response element (PPRE) to regulate the transcription and activation of target genes.^39^ The testicular receptor 4 (TR4) competitively binds to PPRE and inhibits PPARs transcriptional activity, which could be reversed by JAZF‐1 mediated transcriptional repression of TR4.^39^ Furthermore, JAZF‐1 induces carnitines palmitoyl transferase‐1a expression in the liver by enhancing hepatic PPARs.^18^ After activation of PPARs, visfatin transcription is indirectly promoted by JAZF‐1, which plays an important role in insulin sensitivity.^39^

Although above, several limitations of this study should be acknowledged. First, Treg cells resident in different non‐lymphoid tissues exhibit tissue‐specific properties, while our study focused on Tregs differentiation in VAT.^40^ Second, it is well‐established that a large fraction of VAT Treg cells express the IL‐33 receptor ST2 as well as KLRG1 and CCR2, especially in male mice. An additional subset of VAT Treg cells is characterized by a naive‐like phenotype, expressing CD73 and TCF‐1,^41^ while the current study assessed Treg differentiation in VAT using CD4^+^, CD25^+^ and FOXP3^+^ antibodies. Finally, the IgG immunoprecipitation for negative control in Co‐IP was not applied, and the interactions between JAZF‐1 and PPAR‐γ was restricted.

## 5 CONCLUSION

In summary, this study found that JAZF‐1 inhibits TC, TG and GLU, and JAZF‐1 and PPAR‐γ activation promote Treg differentiation. Moreover, JAZF‐1 and PPAR‐γ activation inhibited TNF‐α, IL‐1β and IL‐6, and promoted IL‐10 and TGF‐β expression. Furthermore, a potential synergistic effect between JAZF‐1 and PPAR‐γ, which plays a key role in insulin resistance, was observed. Therefore, JAZF‐1 could be explored as a novel therapeutic agent to prevent the progression of insulin resistance.

## AUTHOR CONTRIBUTIONS

**Fanping Meng:** Conceptualization (equal); investigation (equal); methodology (equal); writing – original draft (equal). **Po Hao:** Investigation (equal); methodology (equal); validation (equal); writing – original draft (equal). **Hongxin Du:** Data curation (equal); formal analysis (equal); validation (equal).

## FUNDING INFORMATION

This research was supported by the Chongqing Science and Technology Bureau, China (cstc2019jcyj‐msxmX0122).

## CONFLICT OF INTEREST STATEMENT

The authors declare that there are no competing interests associated with the manuscript.

## Supporting information


Appendix S1.

Appendix S2.

Appendix S3.

Appendix S4.

## Data Availability

The original contribution presented in the study are included in the article, further inquiries can be directed to the corresponding author.
