# Supplementary material for: Regulatory T cells differentiation in visceral adipose tissues contributes to insulin resistance by regulating JAZF‐1/PPAR‐γ pathway
Source: J Cell Mol Med. 2023 Feb 3;27(4):553–62. doi: 10.1111/jcmm.17680 (PMC9930433; doi:10.1111/jcmm.17680)
Supplement: Supplementary file 1 — Appendix S1. [file JCMM-27-553-s001.docx]

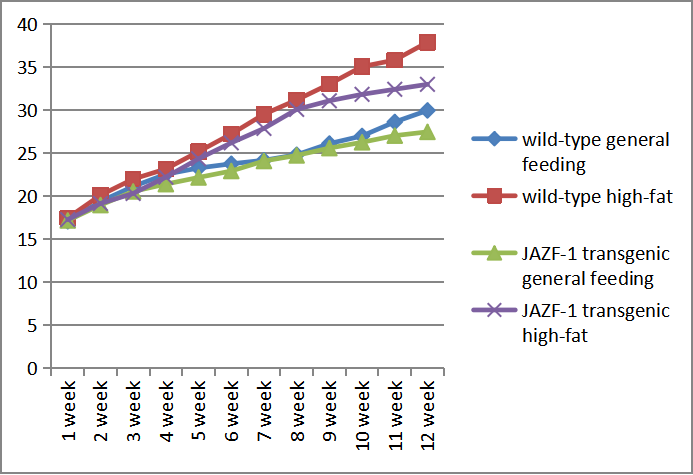


Figure S1. The changes of weight in the wild-type general feeding, wild-type high-fat, JAZF-1 transgenic general feeding, and JAZF-1 transgenic high-fat groups.
