# Supplementary material for: Regulatory T cells differentiation in visceral adipose tissues contributes to insulin resistance by regulating JAZF‐1/PPAR‐γ pathway
Source: J Cell Mol Med. 2023 Feb 3;27(4):553–62. doi: 10.1111/jcmm.17680 (PMC9930433; doi:10.1111/jcmm.17680)
Supplement: Supplementary file 2 — Appendix S2. [file JCMM-27-553-s002.docx]

| 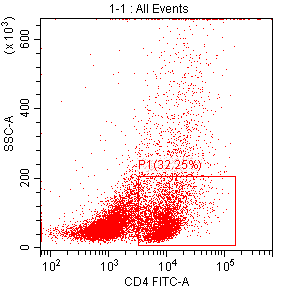 | 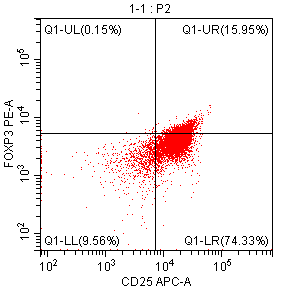 |
| --- | --- |
| Wild-type general feeding group | |
| 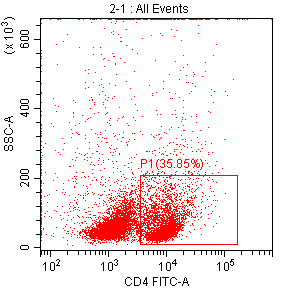 | 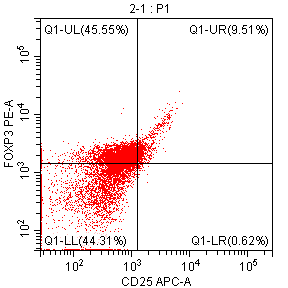 |
| Wild-type high-fat group | |
| 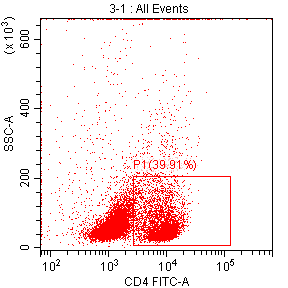 | 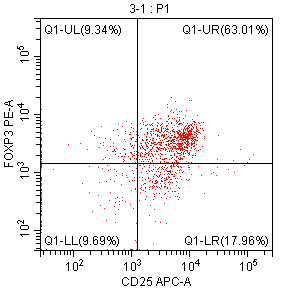 |
| JAZF1 transgenic general feeding group | |
| 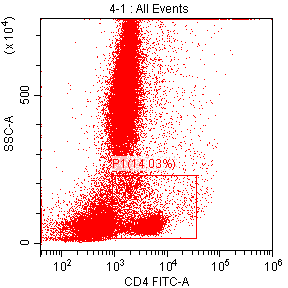 | 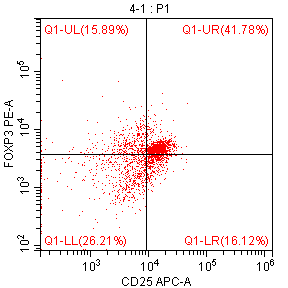 |
| JAZF1 transgenic high-fat group | |

Figure S1. Treg differentiation in VAT was assessed using CD4+, CD25+, and FOXP3+ antibodies (CD4+, CD25+, and FOXP3+ in wild-type general feeding, wild-type high-fat, JAZF-1 transgenic general feeding, JAZF-1 transgenic high-fat groups)

| 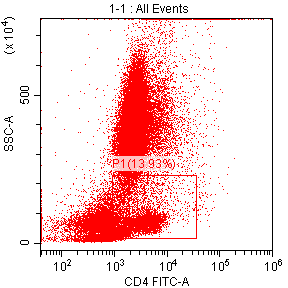 | 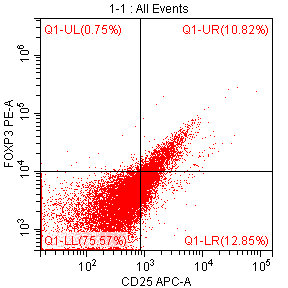 |
| --- | --- |
| Control group | |
| 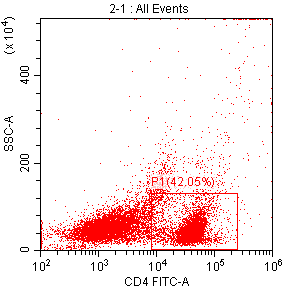 | 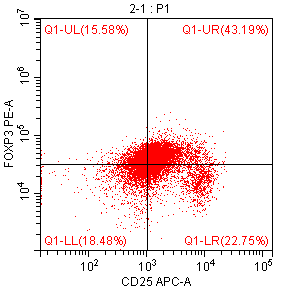 |
| PPAR-γ agonist group | |
| 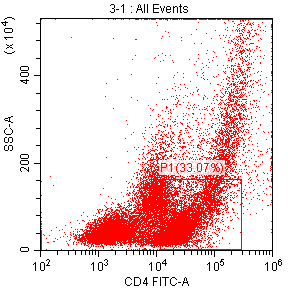 | 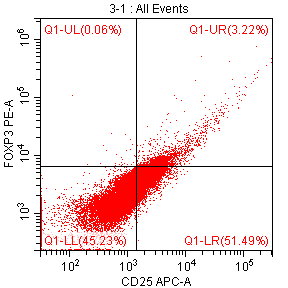 |
| PPAR-γ inhibitor group | |

Figure S2. Treg differentiation in VAT was assessed using CD4+, CD25+, and FOXP3+ antibodies (CD4+, CD25+, and FOXP3+ in control, PPAR-γ agonist, and PPAR-γ inhibitor groups)

| 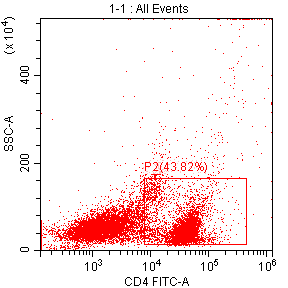 | 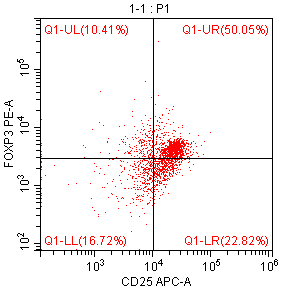 |
| --- | --- |
| General feeding plus normal saline group | |
| 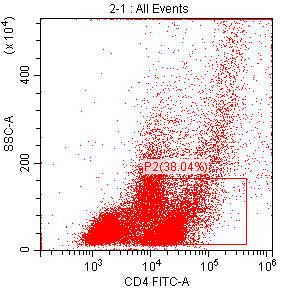 | 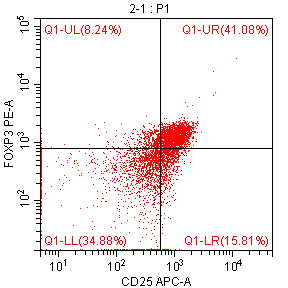 |
| High-fat plus normal saline group | |
| 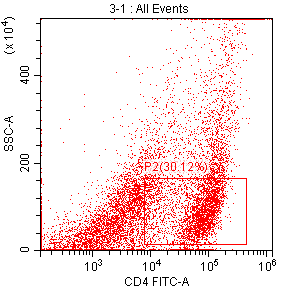 | 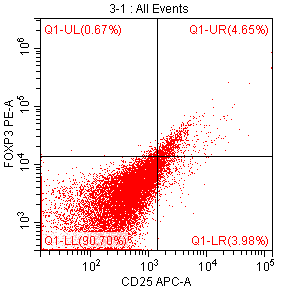 |
| General feeding plus PPAR-γ inhibitor group | |
| 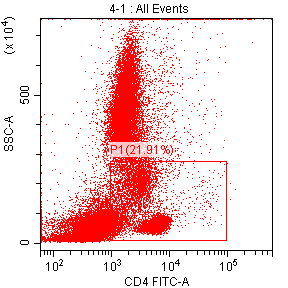 | 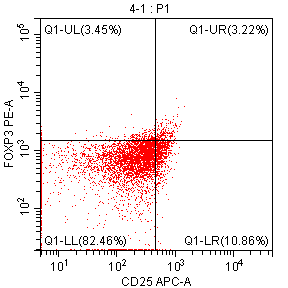 |
| High-fat plus PPAR-γ inhibitor group | |

Figure S3. Treg differentiation in VAT was assessed using CD4+, CD25+, and FOXP3+ antibodies (CD4+, CD25+, and FOXP3+ in general feeding plus normal saline, high-fat plus normal saline, general feeding plus PPAR-γ inhibitor, and high-fat plus PPAR-γ inhibitor groups)
