# Supplementary material for: Regulatory T cells differentiation in visceral adipose tissues contributes to insulin resistance by regulating JAZF‐1/PPAR‐γ pathway
Source: J Cell Mol Med. 2023 Feb 3;27(4):553–62. doi: 10.1111/jcmm.17680 (PMC9930433; doi:10.1111/jcmm.17680)
Supplement: Supplementary file 3 — Appendix S3. [file JCMM-27-553-s003.docx]

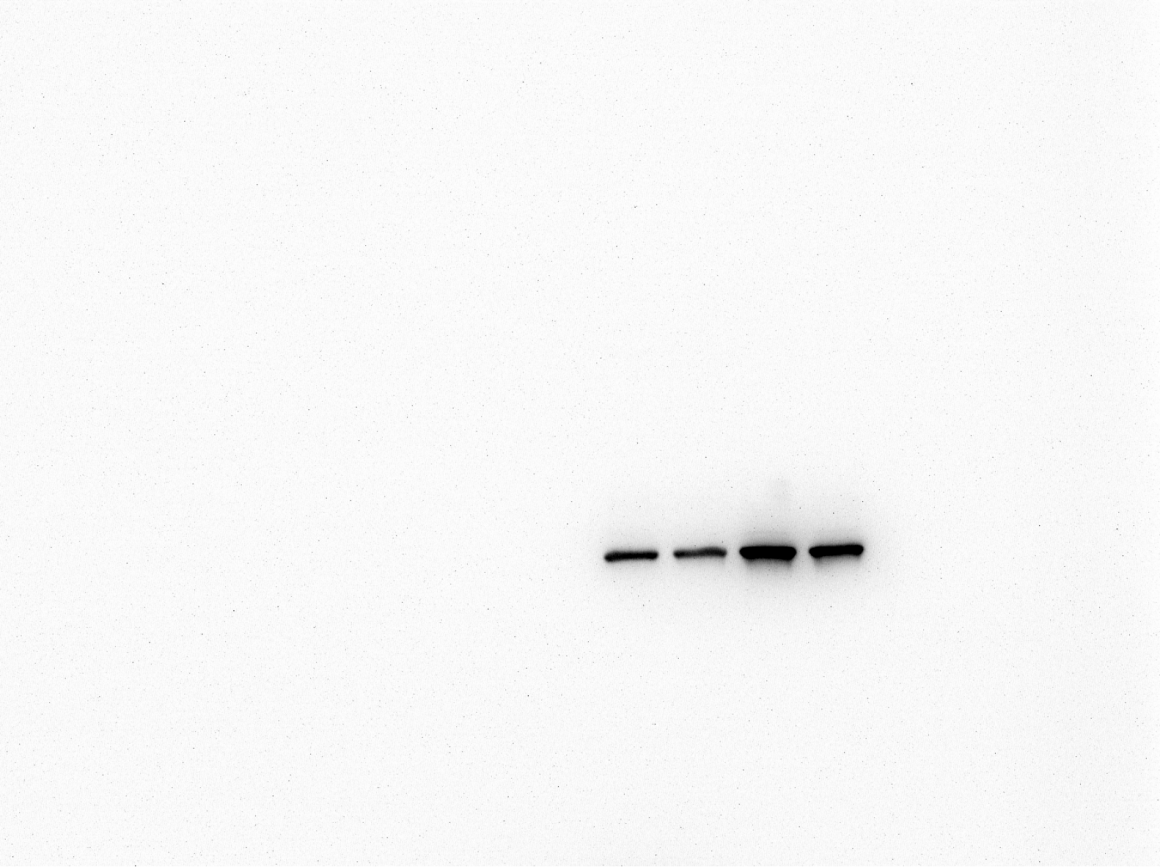


Figure S1. The mRNA and protein expression levels of JAZF-1 in wild-type general feeding, wild-type high-fat, JAZF-1 transgenic general feeding, JAZF-1 transgenic high-fat groups (in accordance with the order of protein expression)


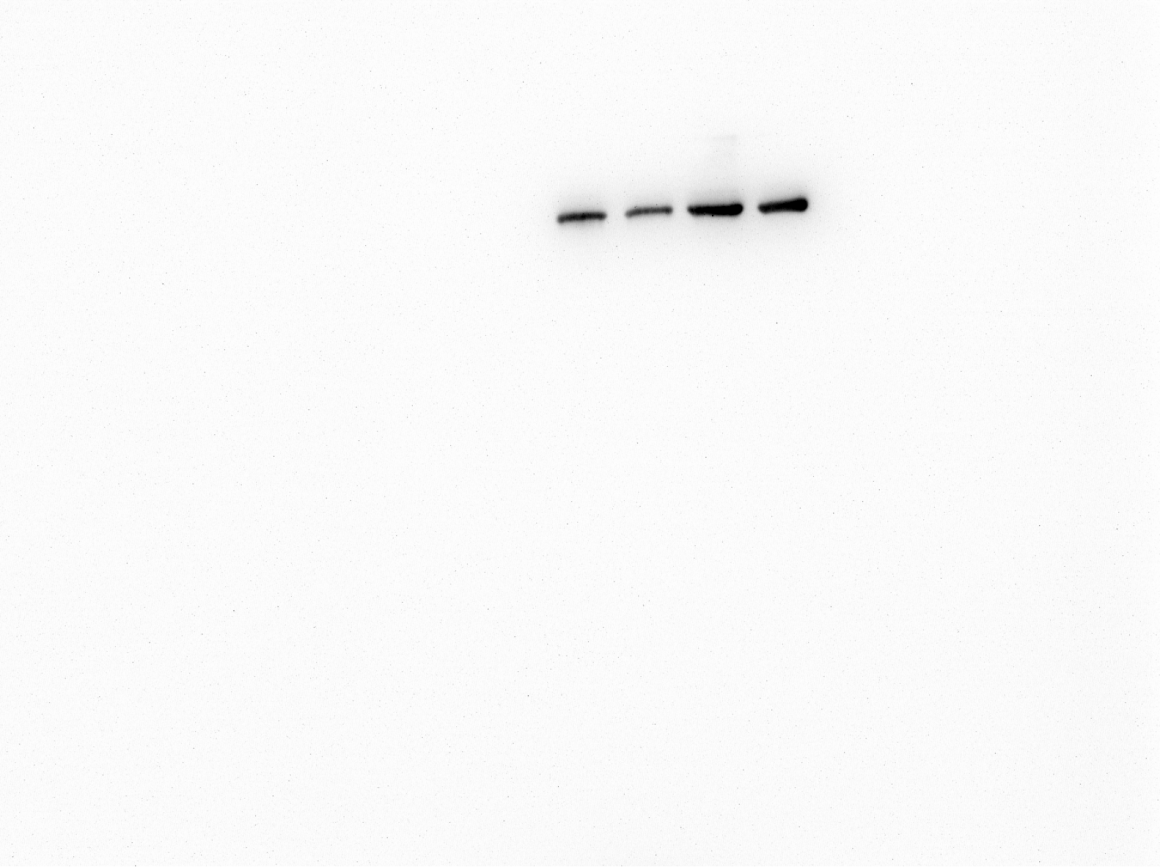


Figure S2. The mRNA and protein expression levels of PPAR-γ in wild-type general feeding, wild-type high-fat, JAZF-1 transgenic general feeding, JAZF-1 transgenic high-fat groups (in accordance with the order of protein expression)


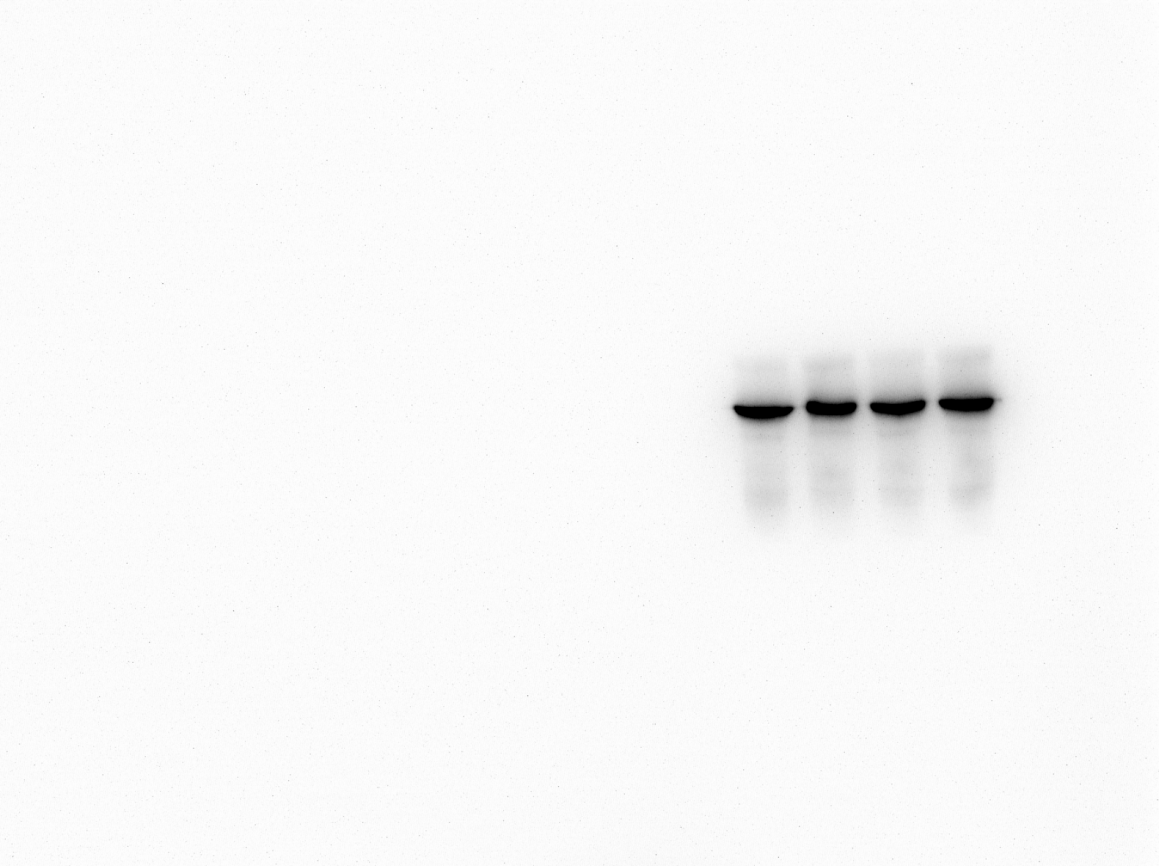


Figure S3. The mRNA and protein expression levels of β-actin in wild-type general feeding, wild-type high-fat, JAZF-1 transgenic general feeding, JAZF-1 transgenic high-fat groups (in accordance with the order of protein expression)


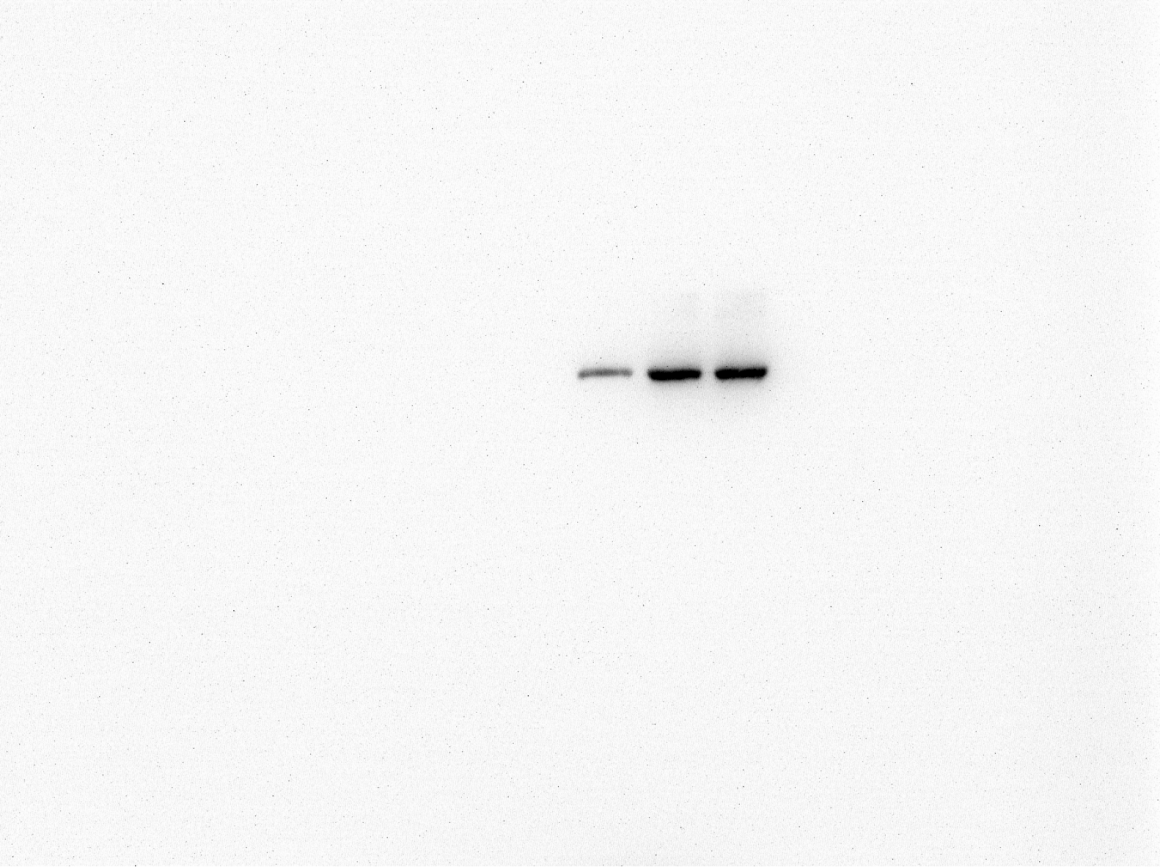


Figure S4. The mRNA and protein expression levels of JAZF-1 in control, PPAR-γ agonist, and PPAR-γ inhibitor groups (in accordance with the order of protein expression)


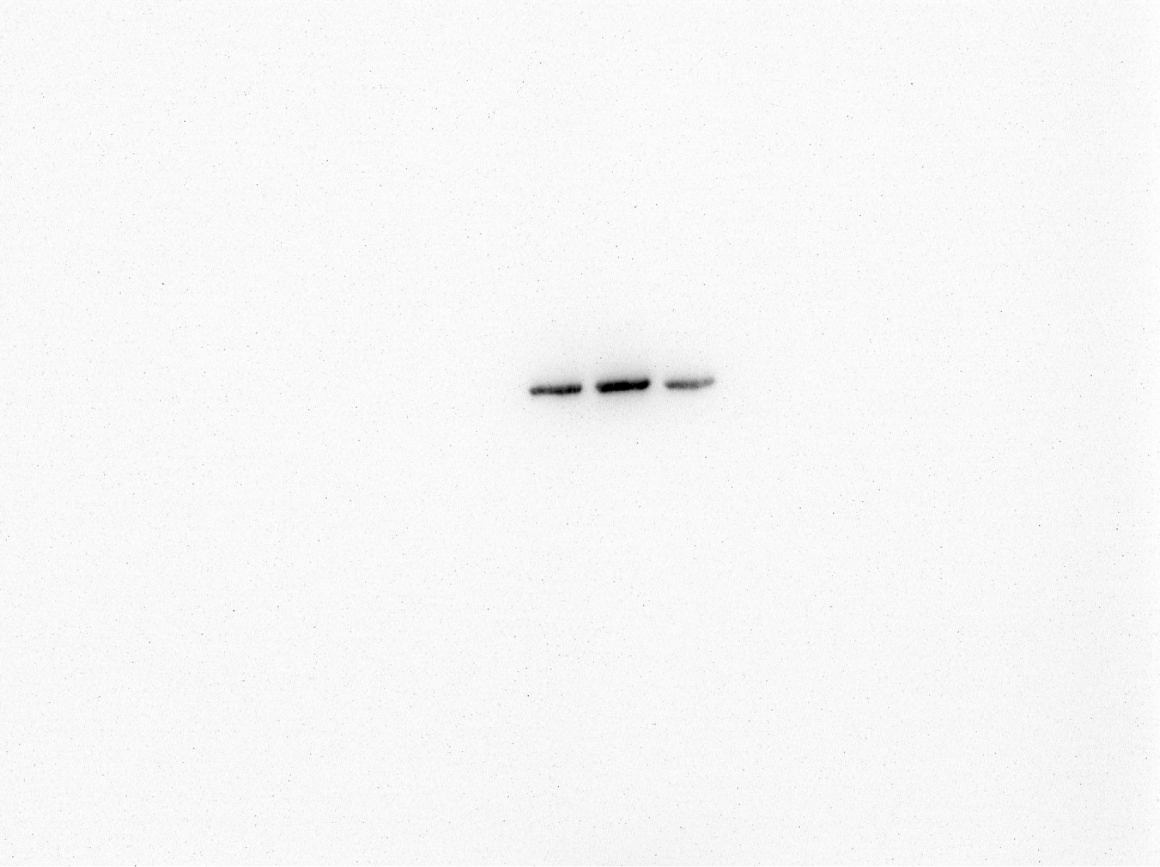


Figure S5. The mRNA and protein expression levels of PPAR-γ in control, PPAR-γ agonist, and PPAR-γ inhibitor groups (in accordance with the order of protein expression)


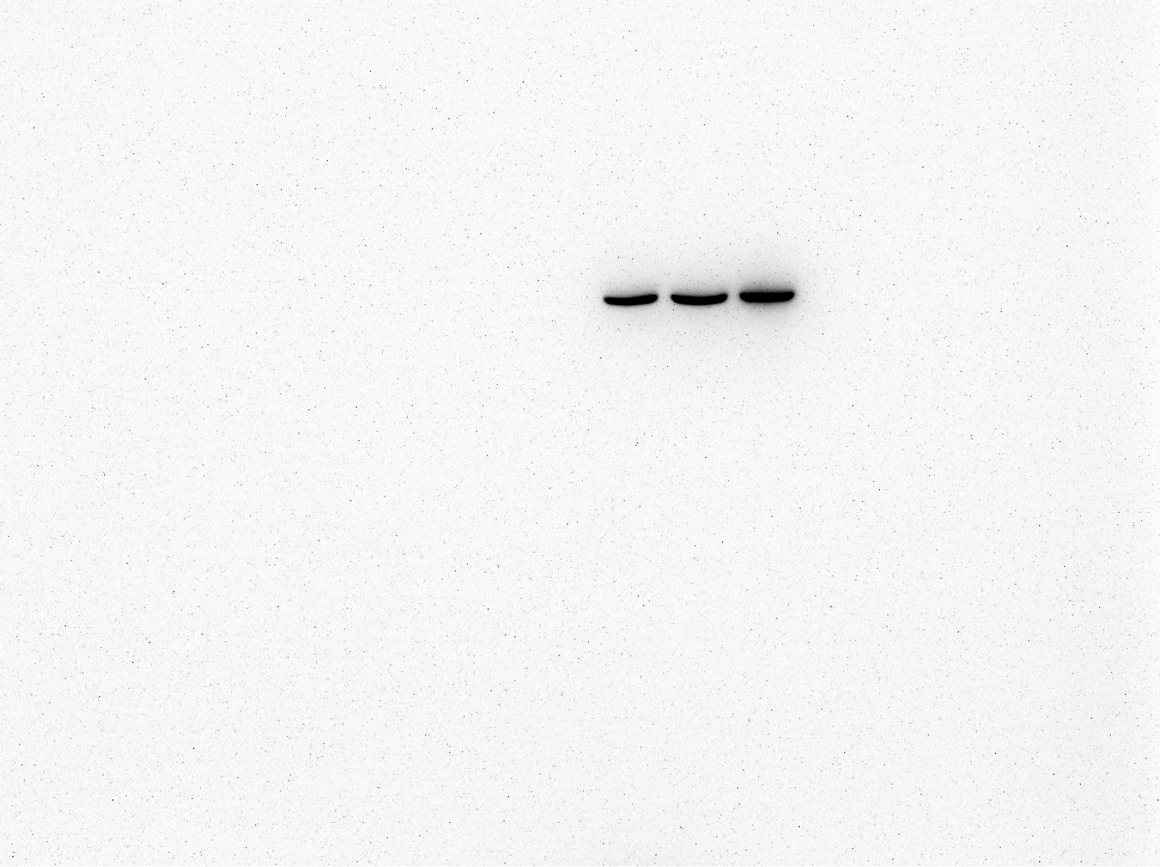


Figure S6. The mRNA and protein expression levels of β-actin in control, PPAR-γ agonist, and PPAR-γ inhibitor groups (in accordance with the order of protein expression)


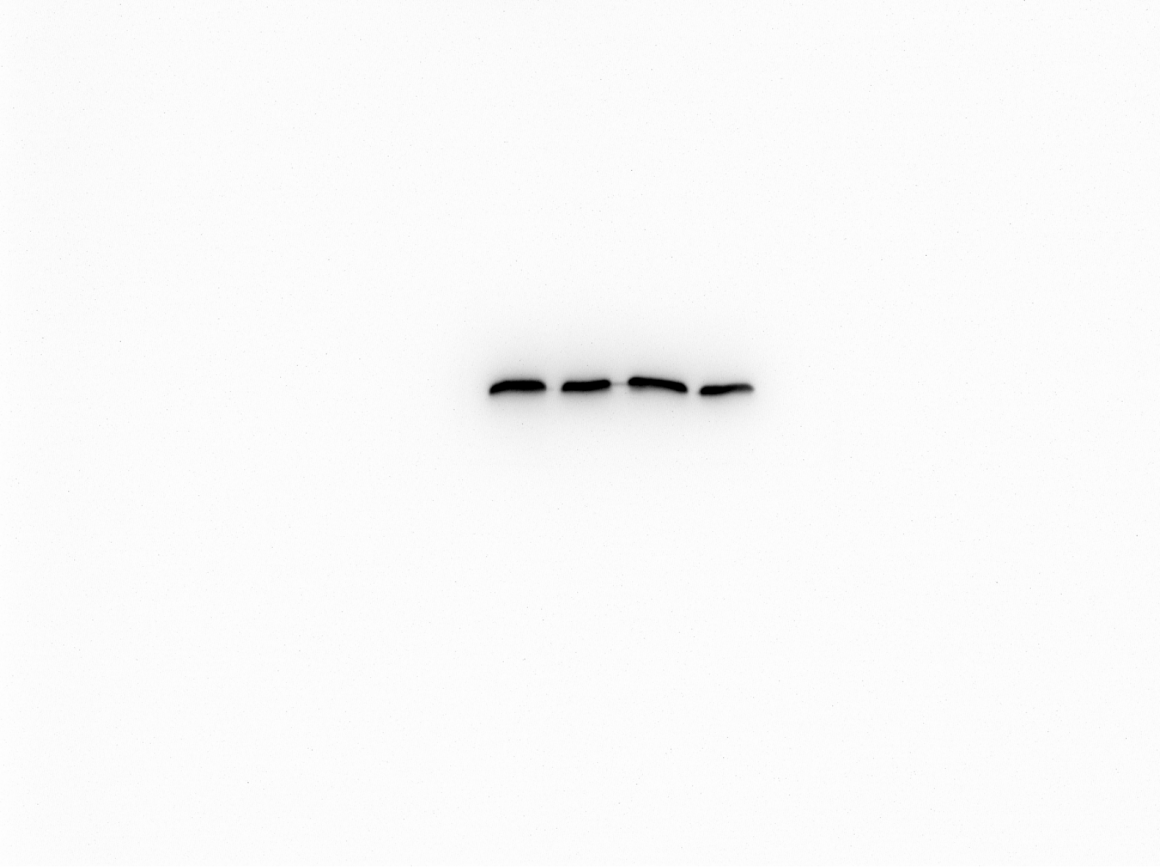


Figure S7. The mRNA and protein expression levels of JAZF-1 in general feeding plus normal saline, high-fat plus normal saline, general feeding plus PPAR-γ inhibitor, and high-fat plus PPAR-γ inhibitor groups (in accordance with the order of protein expression)


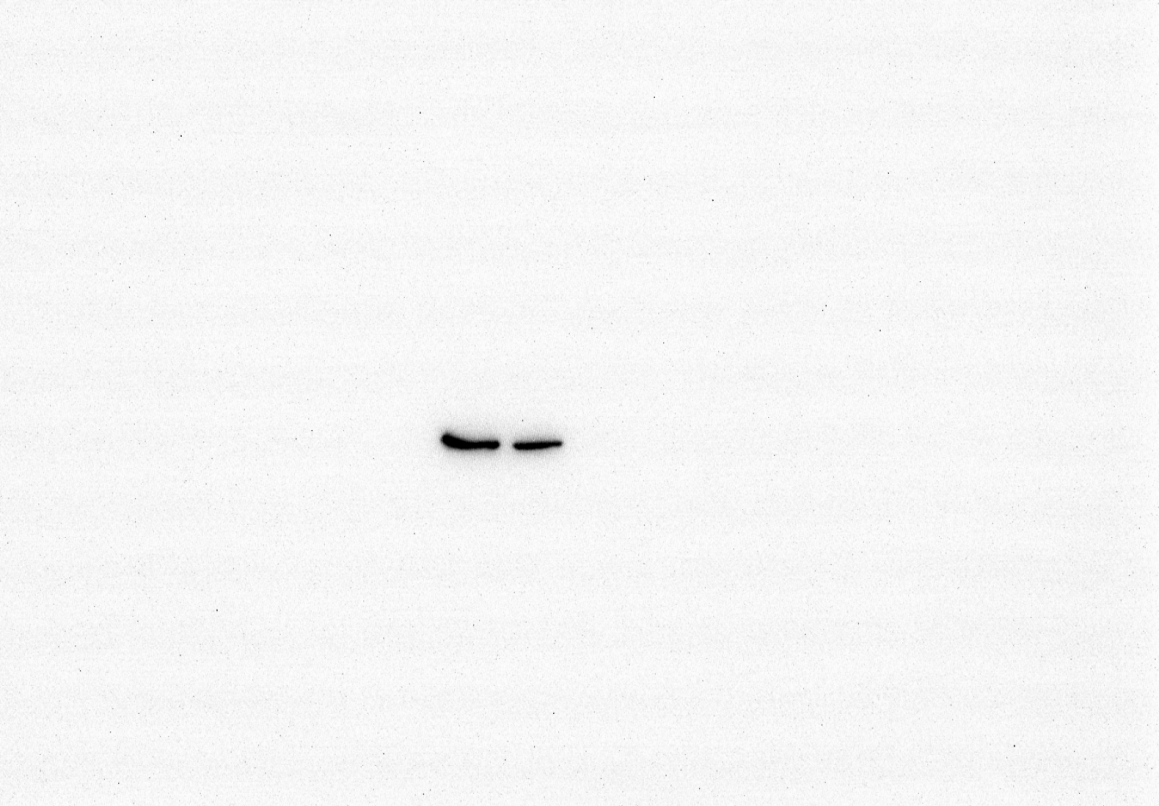


Figure S8. The mRNA and protein expression levels of PPAR-γ in general feeding plus normal saline, high-fat plus normal saline, general feeding plus PPAR-γ inhibitor, and high-fat plus PPAR-γ inhibitor groups (in accordance with the order of protein expression)


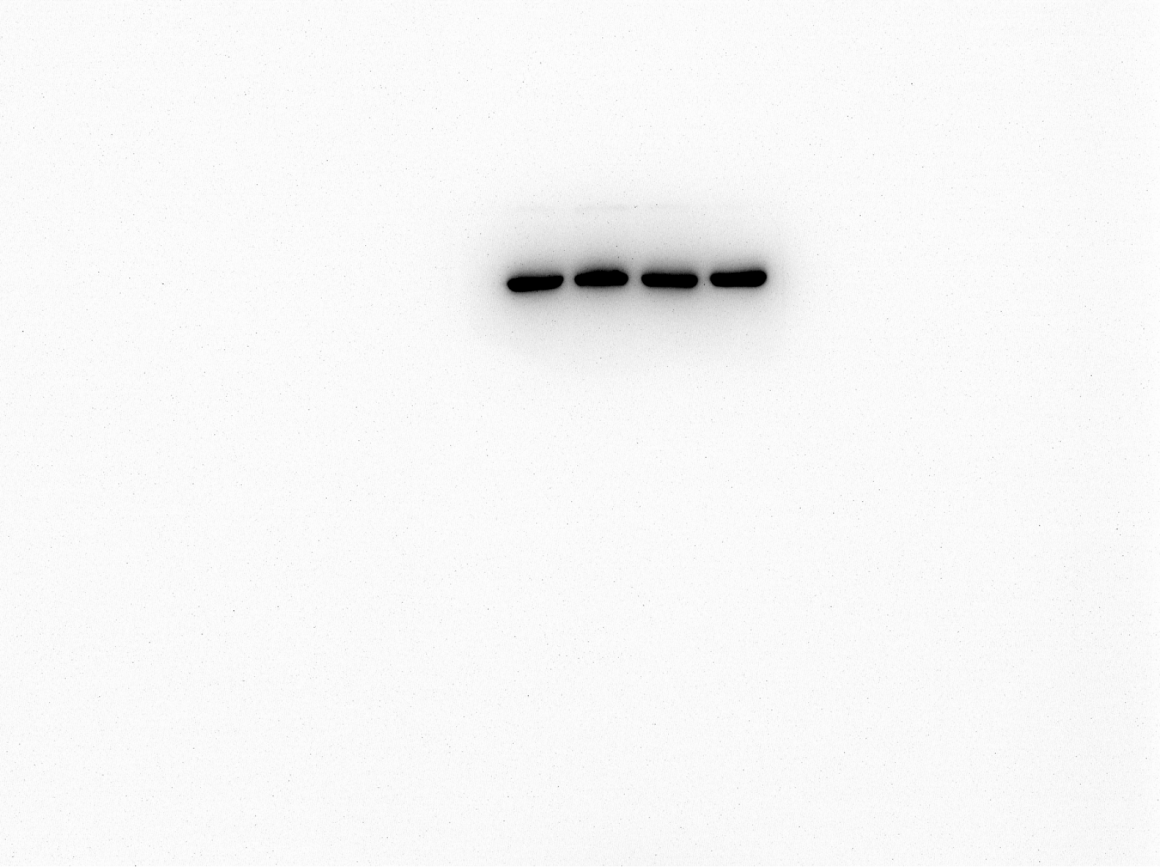


Figure S9. The mRNA and protein expression levels of β-actin in general feeding plus normal saline, high-fat plus normal saline, general feeding plus PPAR-γ inhibitor, and high-fat plus PPAR-γ inhibitor groups (in accordance with the order of protein expression)
