# Supplementary material for: Regulatory T cells differentiation in visceral adipose tissues contributes to insulin resistance by regulating JAZF‐1/PPAR‐γ pathway
Source: J Cell Mol Med. 2023 Feb 3;27(4):553–62. doi: 10.1111/jcmm.17680 (PMC9930433; doi:10.1111/jcmm.17680)
Supplement: Supplementary file 4 — Appendix S4. [file JCMM-27-553-s004.docx]

**Table S1. The expression of JAZF1 and PPARγprotein**

|  | **JAZF1- PPARγ-** | **JAZF1+ PPARγ-** | **JAZF1- PPARγ+** | **JAZF1+ PPARγ+** |
| --- | --- | --- | --- | --- |
| JAZF1 | 0 | 17695 | 0 | 15144 |
| JAZF1 | 0 | 17202 | 0 | 14036 |
| JAZF1 | 0 | 17375 | 0 | 14544 |
| PPARγ | 0 | 0 | 10704 | 12946 |
| PPARγ | 0 | 0 | 10394 | 12690 |
| PPARγ | 0 | 0 | 10031 | 12247 |
| β-actin | 21444 | 20073 | 18417 | 18506 |
| β-actin | 21421 | 19580 | 18247 | 18651 |
| β-actin | 21350 | 20452 | 18762 | 18907 |

**Table S2. Relative protein expression of JAZF1 and PPARγ**

|  | **JAZF1- PPARγ-** | **JAZF1+ PPARγ-** | **JAZF1- PPARγ+** | **JAZF1+ PPARγ+** |
| --- | --- | --- | --- | --- |
| JAZF1 | 0.00 | 0.83 | 0.00 | 0.71 |
| JAZF1 | 0.00 | 0.80 | 0.00 | 0.66 |
| JAZF1 | 0.00 | 0.81 | 0.00 | 0.68 |
| PPARγ | 0.00 | 0.00 | 0.50 | 0.60 |
| PPARγ | 0.00 | 0.00 | 0.49 | 0.59 |
| PPARγ | 0.00 | 0.00 | 0.47 | 0.57 |
| β-actin | 1.00 | 0.94 | 0.86 | 0.86 |
| β-actin | 1.00 | 0.91 | 0.85 | 0.87 |
| β-actin | 1.00 | 0.96 | 0.88 | 0.89 |
